# Artificial Theta Stimulation Impairs Encoding of Contextual Fear Memory

**DOI:** 10.1371/journal.pone.0048506

**Published:** 2012-11-01

**Authors:** Arto Lipponen, Bisrat T. Woldemichael, Kestutis Gurevicius, Heikki Tanila

**Affiliations:** 1 A. I. Virtanen Institute, University of Eastern Finland, Kuopio, Finland; 2 Department of Neurology, Kuopio University Hospital, Kuopio, Finland; IRB Barcelona, Parc Cientific de Barcelona and CIBERNED (ISCIII), University of Barcelona, Spain

## Abstract

Several experiments have demonstrated an intimate relationship between hippocampal theta rhythm (4–12 Hz) and memory. Lesioning the medial septum or fimbria-fornix, a fiber track connecting the hippocampus and the medial septum, abolishes the theta rhythm and results in a severe impairment in declarative memory. To assess whether there is a causal relationship between hippocampal theta and memory formation we investigated whether restoration of hippocampal theta by electrical stimulation during the encoding phase also restores fimbria-fornix lesion induced memory deficit in rats in the fear conditioning paradigm. Male Wistar rats underwent sham or fimbria-fornix lesion operation. Stimulation electrodes were implanted in the ventral hippocampal commissure and recording electrodes in the septal hippocampus. Artificial theta stimulation of 8 Hz was delivered during 3-min free exploration of the test cage in half of the rats before aversive conditioning with three foot shocks during 2 min. Memory was assessed by total freezing time in the same environment 24 h and 28 h after fear conditioning, and in an intervening test session in a different context. As expected, fimbria-fornix lesion impaired fear memory and dramatically attenuated hippocampal theta power. Artificial theta stimulation produced continuous theta oscillations that were almost similar to endogenous theta rhythm in amplitude and frequency. However, contrary to our predictions, artificial theta stimulation impaired conditioned fear response in both sham and fimbria-fornix lesioned animals. These data suggest that restoration of theta oscillation per se is not sufficient to support memory encoding after fimbria-fornix lesion and that universal theta oscillation in the hippocampus with a fixed frequency may actually impair memory.

## Introduction

Hippocampal theta rhythm (4–12 Hz) is one of the most regular rhythms of the brain and it has been associated with a variety of different cognitive functions, especially in memory functions related to the hippocampus (see reviews [1]–[7]). The link between hippocampal theta and spatial memory is based on the observations that medial septum lesion that abolishes hippocampal theta also impairs spatial memory [8]. In addition, transection of the fimbria-fornix conveying projections between subcortical areas and the hippocampus through the medial septum [9], disrupts the hippocampal theta rhythm [10] and leads to memory impairment [11]. One explanation to the dramatic effects of medial septal lesion or fimbria-fornix transections is that both lesions disrupt all neurotransmitter projections from the medial septum to the hippocampus, traveling in fimbria-fornix including glutamatergic [12], cholinergic [13] and GABAergic [14] connections. However, a limitation of lesion studies is that they cannot unambiguously distinguish between the loss of hippocampal theta *per se* and the tissue damage that was induced to wipe out theta as the cause of the ensuing memory deficit.

An alternative approach to assess the role of the theta rhythm in memory is to deliver local electrical stimulation into the brain tissue, either to mimic or to activate the naturally occurring hippocampal theta, and to observe potential improvement in hippocampal-dependent memory functions. The theta rhythm is an intrinsic feature of hippocampal neural networks. Even if the exact cellular mechanisms of hippocampal theta are under a debate, hippocampal slice studies have shown single hippocampal neurons to resonate [15] and fire rhythmically [16] at the theta frequency. As a consequence, the hippocampus is able to generate theta oscillation intrinsically [17]. Therefore, electrical stimulation of the hippocampal networks is able to produce oscillations mimicking naturally occurring theta oscillation [15], [18], [19]. Some clinical studies have shown memory enhancement by electrical stimulation of brain structures intimately connected to the hippocampus [20]–[23], see also review [24]. However, those studies used brain stimulation to induce a general cortical activation [19], [25], and did not specifically address the role of hippocampal theta (or any other specific oscillation) in memory *per se*. To this end, a more specific behavior-stimulation paradigm is required.

Only a few studies have actually considered the possibility to restore initial memory functions by ATS (artificial theta stimulation) in animals in which theta has been abolished by lesioning or by pharmacological inactivation. One study [26] reported improvemed performance in a working memory variation of the Morris water maze by ATS in fimbria-fornix lesioned animals. However, a positive response to ATS was obtained in an elegant experiment in which hippocampal theta rhythm was first temporally blocked by a local anaesthetic (tetracaine) injection into the medial septum [27] and then restored by electrical stimulation mimicking the intact theta activity in the supramammillary area. The treatment was able to restore initial learning in the Morris water maze [18]. In a similar experimental setup, in which hippocampal theta was first blocked by the GABA agonist muscimol injection into the medial septum [28], electrical stimulation of the fimbria-fornix restored memory in the Morris swim task. However, single-pulse theta stimulation alone proved to be ineffective while a theta-burst pattern that produced theta-gamma co-modulation resulted in significant memory improvement [19]. One limitation of the water maze task in this context is that each trial involves both memory encoding and retrieval. To delve into the mechanisms of ATS it would be essential to make a distinction between the two processes.

A well established model to study hippocampal memory functions is the contextual fear conditioning paradigm, where an animal receives unsignaled foot shock(s) in a novel experimental context. When the animal is placed back into the familiar context it expresses characteristic freezing behavior related to aversive stimuli [29], [30]. In contextual fear conditioning the amygdala has been shown to be the key neural structure for making the association between a neutral cue and the aversive foot shock (see review [31]–[34]), while the hippocampus is thought to co-operate by processing the contextual cues of environment. Accordingly, contextual fear conditioning is disrupted by hippocampal lesions (for instance see [35]–[39]) and by fimbria-fornix lesion (FFX) [39]–[41]. The advantage of this task is that is has distinct phases for memory encoding, consolidation and retrieval. In addition, a long-lasting memory trace can be formed in a single training session.

Synchronized brain oscillations are supposed to work as the mechanism to enable network communication underlying memory encoding and retrieval at the systems level [42]. The hippocampus and amygdala have been demonstrated to interact during fear conditioning via theta phase synchronization that occurs as a response to conditioned fear stimuli and exists during memory consolidation and reconsolidation [43], [44]. At the cellular and molecular level, disruption of normal protein synthesis either in the hippocampus [45] or amygdale [46]during consolidation or reconsolidation leads impaired fear memory [47], [48]. Egr-1 (early growth response protein 1, also called as zif268), a nuclear protein functioning as a transcriptional regulator (see review [49]), plays a crucial role in fear conditioning. Egr-1 is involved not only during encoding [50] of fear memory but also during consolidation [51]–[53] and reconsolidation [54], [55] in both the hippocampus [50], [53], [54], [56] and the amygdala [51], [53], [55], [57].

This experiment set out to test whether restoring hippocampal theta activity of memory impaired FFX lesioned animals could specifically improve the encoding of contextual memories. In addition, we wanted also to assess whether restoration of hippocampal theta also influences learning-associated molecular changes (Egr1) in the hippocampus and amygdala.

## Methods

### Ethics Statement

All experiments were conducted in accordance with the guidelines of the Council of Europe and approved by the State Provincial Office of Eastern Finland.

The subjects were male Wistar rats from the Laboratory Animal Center at University of Eastern Finland ranging 13–17 weeks in age and 375±16.4 g (mean ± SEM) in weight at the time of the surgery. Animals were caged after operation individually in a controlled environment with temperature kept at +21°C and light on from 7∶00 to 19∶00, and water and food available *ad libitum.* In total, 24 animals were used in this experiment. The animals were divided into four groups depending on the lesioning type (either fimbria-fornix (FFX) or sham lesion) and the artificial theta stimulation (ATS) treatment (either ATS or no ATS).

Surgery was performed under isoflurane gas anaesthesia, with the induction airflow at 450 l/min (4.5%) and maintenance at 250 l/min (2.1%). Animals were chronically implanted with bipolar electrodes (Formwar® insulated stainless steel wire, diameter 100 µm, California Fine Wire Company Co, Grover Beach, CA, USA) with vertical tip separation of 600 µm in the following brain coordinates: a bipolar recording electrode in the right septal hippocampus at AP −3.8 mm (from bregma), ML 2.6 mm (from the midline), and DV 3.2–3.4 mm (from the dura) and a bipolar stimulation electrode in the right ventral hippocampal commisure at AP −1.8 mm, ML 1.8 mm and DV 4.0 mm. In addition, four screw electrodes were fixed on the cranium on the left and the right frontal and parietal cortices. These screws served also as the anchors for dental acrylic cement and the connector.

**Figure 1:**
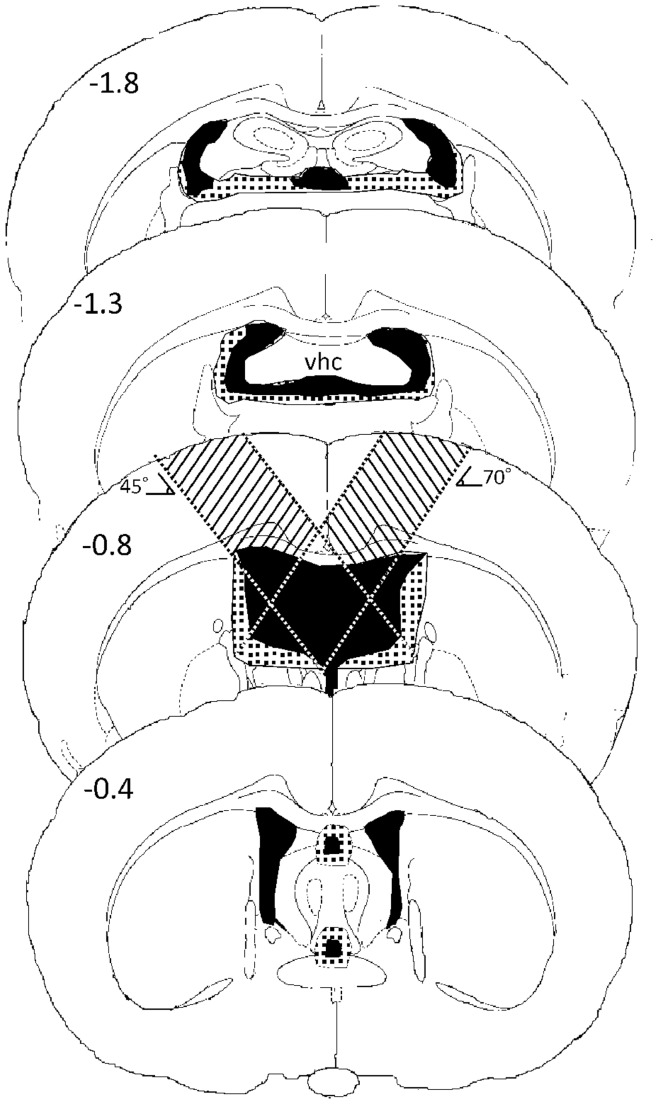
Successive coronal sections through the rat brain illustrating the extent and location of the smallest (black) and largest (dotted) fimbria-fornix lesion. Numbers show the A-P distance from bregma. The coronal section 0.8 mm from bregma also illustrates the knife lesioning technique used; note that lesioning both in 45° and 70° angles were made bilaterally. VHC, ventral hippocampal commissure.

The fimbria-fornix lesion (FFX) was done bilaterally by inserting custom made lesioning knives (from a razor blade, tip width ∼2 mm) through craniotomies at the following coordinates: AP −0.92, ML +1.20–4.00 and AP −0.92, ML −1.20– (−4.00). The knife was attached to a custom made handle attached to the streotaxic arm which allowed us to insert the knife first at a 45° angle and then at a 70° angle related to skull into 5.5 mm from dura. Then the knife was moved back and forth between the ML coordinates to sever the tissue. In sham operation only craniotomies were made without damaging the dura (Fig. 1).

After the surgery, the rat received carprofen (5 mg/kg, i.p., Rimadyl®, Vericore, Dundee, UK) for postoperative analgesia. Carprofen continued (5 mg/kg/day) in drinking water max 3 days, and antibiotic powder (bacitrasin 250 IU/g and neomycinsulfate 5 mg/g, Bacibact®, Orion, Finland) was applied, if necessary, onto the wound.

After a recovery period of 7 days the animals were accustomed to the recording daily for 30 minutes during 5 consecutive days in a setup mimicking the actual testing and recording situation to avoid additional freezing caused by novelty of EEG recording itself. The contextual fear conditioning paradigm was modified from [39]. The rat was placed in the testing cage (the preshock context, Context A) for 3 min during which artificial theta stimulation was delivered. Right after the ATS period three unsignaled foot shocks were delivered (0.7–0.8 mA, pulse duration 100 ms, ISI 64 s). The pulses were delivered through a metal grid floor and generated by a Grass 88 stimulator connected with a stimulus isolator unit (Grass medical Instruments, Quincy, MA, USA).

**Figure 2:**
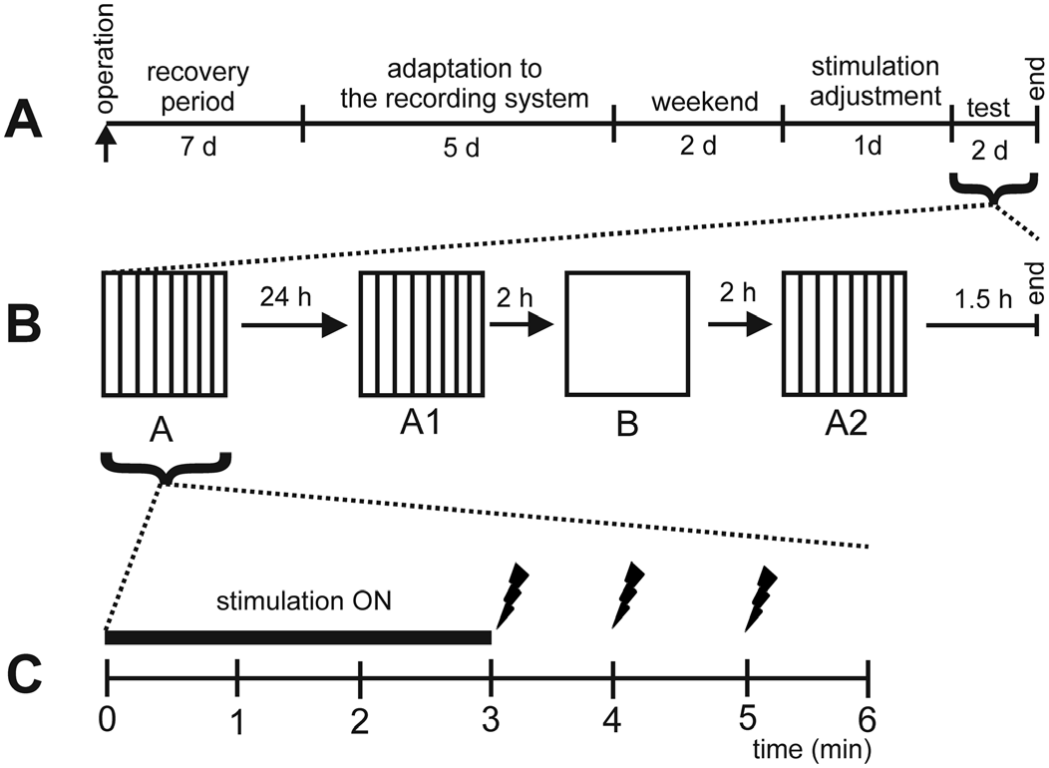
Schematic illustration of the experiment in the time frame of the entire experiment (A), the test session (B), and initial fear conditioning (C). After surgical operations and adaptation period the animals underwent testing phase in which the driving current was adjusted. In the test phase, first, in Context A baseline freezing (preshock) was recorded for 3 min with artificial theta stimulation delivery, which was then followed by fear conditioning with three repeated foot shocks. Fear memory was tested by measuring freezing time first 24 h later in the same familiar context (now marked as Context A1), then 26 h later in a totally novel context (B), and finally 28 h later again in the familiar context (A2).

Twenty-four hours after the conditioning the animal was returned to the conditioning cage (Context A1) for behavioral follow-up for 5 minutes while no shock was delivered, and then returned to the home cage. Two hours later the animal was placed into a totally novel cage (Context B) for 5 minutes. Finally, two hours after the exposure to Context B the animal was placed again in the original conditioning cage (Context A2). During these 5-min observation periods the behavior of the rat was video recorded. The recordings were used for offline analysis of freezing (“absence of any visible movement except for movement of the whiskers and respiration related movements, while the animal is in the crouching position” [29]. Time spent in freezing was recorded and expressed as percent of total time in the context (5 min) (Fig. 2).

EEG was recorded during the first 3 min before the shock delivery in the conditioning cage and during the entire 5-min observation periods in Contexts A1, B and A2. Two hippocampal and three cortical channels were recorded and referred to the left frontal screw, which also worked as the animal ground. The connector was attached to a custom made preamplifier and the signal was further amplified with an AC amplifier (gain 500, A-M Systems Inc., Sequim, WA, USA). Thirty sweeps of 10 s duration were collected and digitized with a sampling frequency of 332 Hz. The signal was band-pass filtered between 1–1000 Hz with the notch filter. The data were acquired by using SciWorks 5.0 program (DataWave Technologies, Loveland, CO, USA). From EEG data seven representative 2-s epochs of movement and freezing were selected. The selected sweeps were filtered (2–48 Hz) using a two-way least-squares FIR filter (the eegfilt.m routine from the EEGLAB toolbox, [58], [59]. The frequency analysis was performed on each individual segment and the FFT (Fast Fourier Transformation) of each segment was used to create the total average. The effect of sham or fimbria-fornix lesioning was analyzed by comparing the average power of theta (4–12 Hz) of these groups during freezing and movement.

Artificial theta stimulation was delivered only in Context A. ATS was delivered for 3 min period before the fear conditioning (unsignaled foot shocks) took place in the same session. It encompassed repeated unipolar pulses of 500 µs with an inter-stimulus interval of 125 ms (8 Hz) mimicking hippocampal theta (4–12 Hz) [1]. It should be noted that upper frequencies in this range (9–12 Hz) overlap with the classic cortical alpha band (9–12 Hz) [60], [61]. The stimuli were generated with a Grass S88 stimulator (Grass medical instruments, Quincy, MA, USA) connected with a stimulus isolator (A360, WPI, Sarasota, FL, USA). The theta driving current was adjusted individually to induce regular hippocampal theta with amplitude matching the mean theta amplitude of a sham operated animals (FFX animals: 99.4±4.0 µA; sham operated animals: 113.8±3.0 µA, mean ± SEM). If we observed epileptiform activity during trial stimulation the animals (n = 2) were excluded from the experiment.

After 90 minutes since the last observation period (in Context A2) the animal received an overdose of medetomidine (0.125 mg/kg) and ketamine (18.75 mg/kg). The electrode locations were marked by passing 1 mA of DC current for 8–10 s through the electrodes. Then the animal was perfused with ice-cold saline for 5 min at 13 ml/min followed by 4% paraformaldehyde solution for 13 min at 13 ml/min. The brain was moved from the skull and left for immersion postfixation for 4 h in 4% PFA and after that in 30% sucrose solution for two days. The brains were stored at −20°C until slicing. Coronal sections (thickness 35 µm) were cut with a freezing slide microtome. The electrode locations were verified from the sections by cresyl violet with Prussian blue staining. In addition, sections collected throughout the hippocampus were stained for acetylcholinesterase (AChE) to verify the extent of the fimbria-fornix lesion. The sections were digitized using an Olympus digital camera with low magnification (2×), and the images were converted to 8-bit gray-scale images using Adobe Photoshop CS4. The density of AChE staining in the CA2/CA3 region of dorsal hippocampus and in the cortex above (from bregma: AP −4.4 mm) were measured using the Photoshop CS4 program. For statistical analysis we compared the density of hippocampal area to the unaffected cortical area [62]–[65].

Egr-1 immunohistochemical analysis was performed on all animals. Three consecutive sections were pretreated in hot 0.05 M sodium citrate solution at pH 6.0 (80°C) for 30 min and then cooled down in 0.1 M sodium phosphate buffer at pH 7.4. Pretreated sections were rinsed three times in Tris Buffered Saline and Triton solution (TBS-T, pH adjusted to 7.6) for 5 minutes. The sections were then incubated in the primary antibody, rabbit anti-Egr, (1∶1 000, Santa Cruz) for overnight at room temperature. Sections were again washed three times in TBS-T then incubated in the secondary antibody, goat anti-rabbit biotinylated (1∶500, Vector). After rinsing three times for 5 min in TBS-T, sections were incubated with StreptAvidin (1∶1 000, GE Healthcare) for 2 h at room temperature. Sections were washed again three times in TBS-T. The sections were then developed using 3.3-diaminobenzidine with nickel ammonium sulfate solution. The reaction was stopped by washing in a sodium phosphate buffer. Sections were then mounted on gelatin-covered slides and cover-slipped.

Egr-1 counts focused on the amygdala and on the septal hippocampal dentate gyrus (DG) because of the low constitutive activity compared to CA1 area. The sparse number of Egr-1 positive cells was counted only from the left hippocampus, as the septal hippocampal electrode located in the right hemisphere might caused an inflammatory reaction which might have influenced Egr-1 expression. The cell count was done on three consecutive hippocampal sections at 5×magnification using a light Olympus microscope fitted with digital camera.

For the amygdala with larger number of Egr-1 positive cells the analysis was accomplished with the aid of Stereo Investigator software (version 5.05.4, Microbrightfield Inc., Magdeburg, Germany). The computer randomly overlaid a 100 µm × 100 µm grid over the drawn contour. This area represents the area associated with each x,y movement and the steps between each counting frame, which had the size of 100 µm ×100 µm.

The EEG data were analyzed with Matlab R2008a, (MathWorks, Natick, MA, USA) software while SPSS 17 for Windows, (SPSS inc. USA) was used for statistical analysis. The graphs were created with GraphPad Prism 5 for Windows, 2009 Software (GraphPad Prism Software Inc. USA).

## Results

### Histological Verification of the FFX Lesion

**Figure 3:**
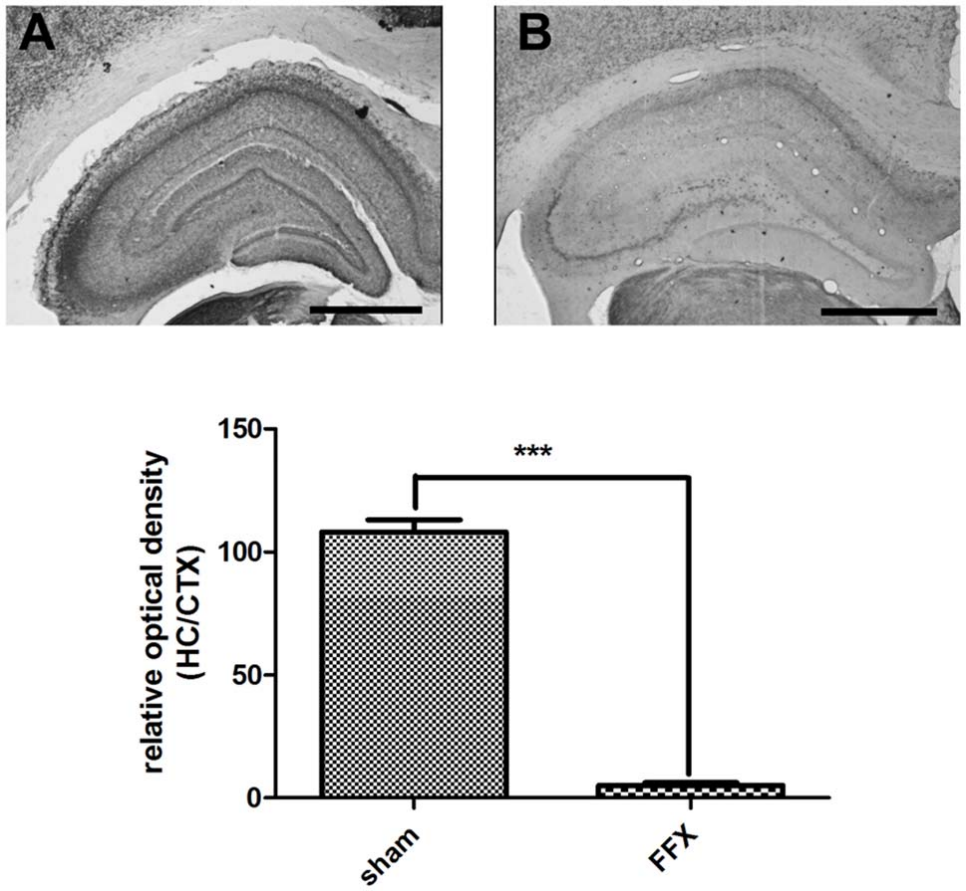
Example of histological slices verifying the effect of fimbria-fornix lesioning on hippocampal cholinergic system. In the fimbria-fornix lesioned animals (B) a substantial loss of cholinergic innervations was revealed by AChE staining compared to sham operated animals (A). The scale bar represents 1.0 mm and the columns and error-bars mean ± SEM, *** *P*<0.001, (sham, n = 12; FFX, n = 12).

Because the fimbria-fornix bundle carries the major cholinergic input to the hippocampus from the medial septum, lesioning this track induces significant cholinergic loss only in the hippocampus [10], [63], [66]. In addition, the extent of cholinergic lesion can be taken as an indicator of the extent of damage to all major septohippocampal pathways including cholinergic, GABAergic and glutaminergic projections. Accordingly, all FFX lesioned rats accepted to this study had a dramatic loss of AChE staining in the hippocampus with deteriorated laminar structure compared to unaffected cortical area (sham 106.8±6.1; FFX 5.1±1.1, P<0.0001) (Fig. 3).

### Loss of Theta Rhythm in FFX Group and its Restoration by Artificial Theta Stimulation

**Figure 4:**
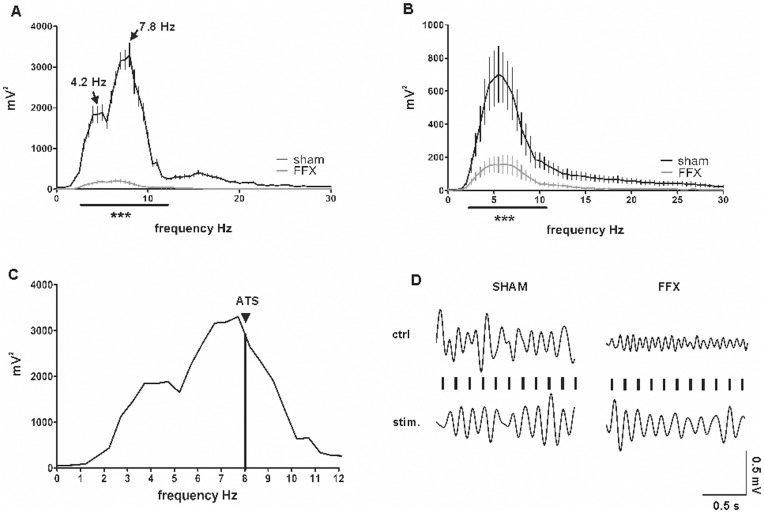
The EEG analysis revealed the effect of the fimbria-fornix lesioning on theta frequency range (4–12 Hz, marked with a horizontal bar) in the septal CA1 area during exploration (A) and freezing (B). The lines represent the spectral analysis of EEG of the sham operated (n = 7) and the FFX (n = 6) group. The error-bars represent SEMs, and asterisks denote frequency bands with significant differences at the level *P*<0.01. (C) Zoomed-in presentation of the same power analysis of sham-operated animals during exploration as in (A). The vertical line denotes the frequency of ATS (8.0 Hz) that was slightly above the mean peak frequency (7.8±0.2 Hz) of sham animals during exploratory behavior. (D) Examples of filtered (2–12 Hz) hippocampal EEG related to exploratory behavior in a sham-operated and a fimbria-fornix lesioned animal during both control situation and artificial theta stimulation (ATS). The occurrence of stimulation pulses is shown on top of the sample EEG sweep.

The electrode locations were confirmed by histology, and only electrodes located in the septal hippocampal CA1 area in layers *stratum oriens - radiatum* were chosen for EEG analysis. The stimulation electrode was confirmed to locate in the ventral hippocampal commissure. In line with the extent of cholinergic depletion, the hippocampal EEG showed a dramatic reduction in hippocampal theta power (4–12 Hz) in the FFX group compared to sham animals. The power spectrum was analyzed with point-by-point t-tests and the results were verified also with Mann-Whitney-test (P<0.001, Fig. 4 A and B). Artificial theta stimulation restored a continuous theta oscillation with a comparable amplitude and frequency as the natural theta (Fig. 4 C and D). The stimulation did not induce any overt behavior on top of rats’ natural exploration and occasional freezing in a novel environment.

### Both FFX and ATS Impair Conditional Freezing

**Figure 5:**
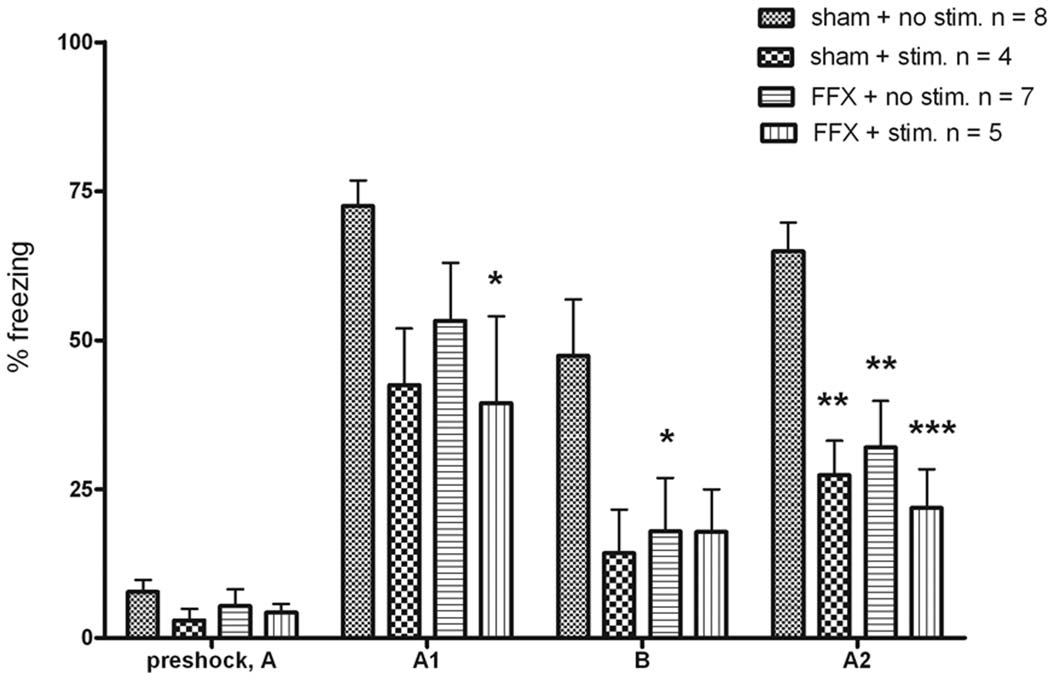
Freezing behavior expressed as percentage of time spent freezing during 5 minutes in the contexts used in the experiment; preshock, the conditioning chamber (A), the novel chamber (B) and re-exposure to the conditioning chamber (A2). Dunnett’s two-sided post-hoc test was used to compare each test group with the non-stimulated sham group as a controlThe columns and error-bars represent mean ± SEM, * *P*<0.05, ** *P*<0.01, *** *P*<0.001.

We used the ANOVA for repeated measures with lesion and stimulation as between-subject factors and contexts (preshock, A1, B and A2) as within-subject factors to test how conditioning itself and the different contexts affect rats’ freezing behavior. This analysis revealed a highly significant overall effect of context (F_3,60_ = 45.8, *P*<0.001). As is evident from Figure 5 and confirmed by a Bonferroni *post-hoc* test for the repeated-measures variable, during preshock animals froze less compared to the other contexts (P<0.002), proving that our fear conditioning test did work. In addition, animals froze less in the novel context (B) and in the re-exposure of conditioned context (A2) than in the conditioned context (A1) (both P<0.003). Although the second re-exposure to the familiar context (A2) induced much less freezing than the first post-shock exposure, animals still froze more in Context A2 than in the novel context B (P = 0.045).

To analyze the effects of lesion and stimulation on freezing time, we ran a two-way ANOVA for each test session. None of the main effects (lesion or stimulation) or their interaction was significant in the baseline freezing before foot shock. However, 24 h after fear conditioning in the shock context Fig. 5, A1the ATS reduced freezing significantly (stimulation vs. no stimulation; F_1,20_ = 5.2, P = 0.03), whereas the main effect of lesion (F_1,20_ = 1.3, P = 0.26) or the lesion by stimulation interaction were nonsignificant (F_1,20_ = 0.7, P = 0.41). When the animals were tested 26 h after the fear conditioning in a totally novel context (Fig. 5, B), neither FF-lesion nor stimulation had a significant effect on the freezing time (P>0.096). After returning the animals to the original context for the second time (Fig. 5, A2) 28 h after the fear conditioning both the lesion (F_1,20_ = 8.1, *P = *0.01) and the ATS (F_1,20_ = 12.5, *P* = 0.002) had a significant reducing effect on the freezing time while their interaction only approached significance (F_1,20_ = 4.1, *P* = 0.056). As a conclusion we can note that note that both FFX and ATS attenuated conditioned freezing to the same extent. However, combination of FFX and ATS had only little additive effect over either treatment alone.

### Decreased Egr-1 Protein Expression in FFX Animals

**Figure 6:**
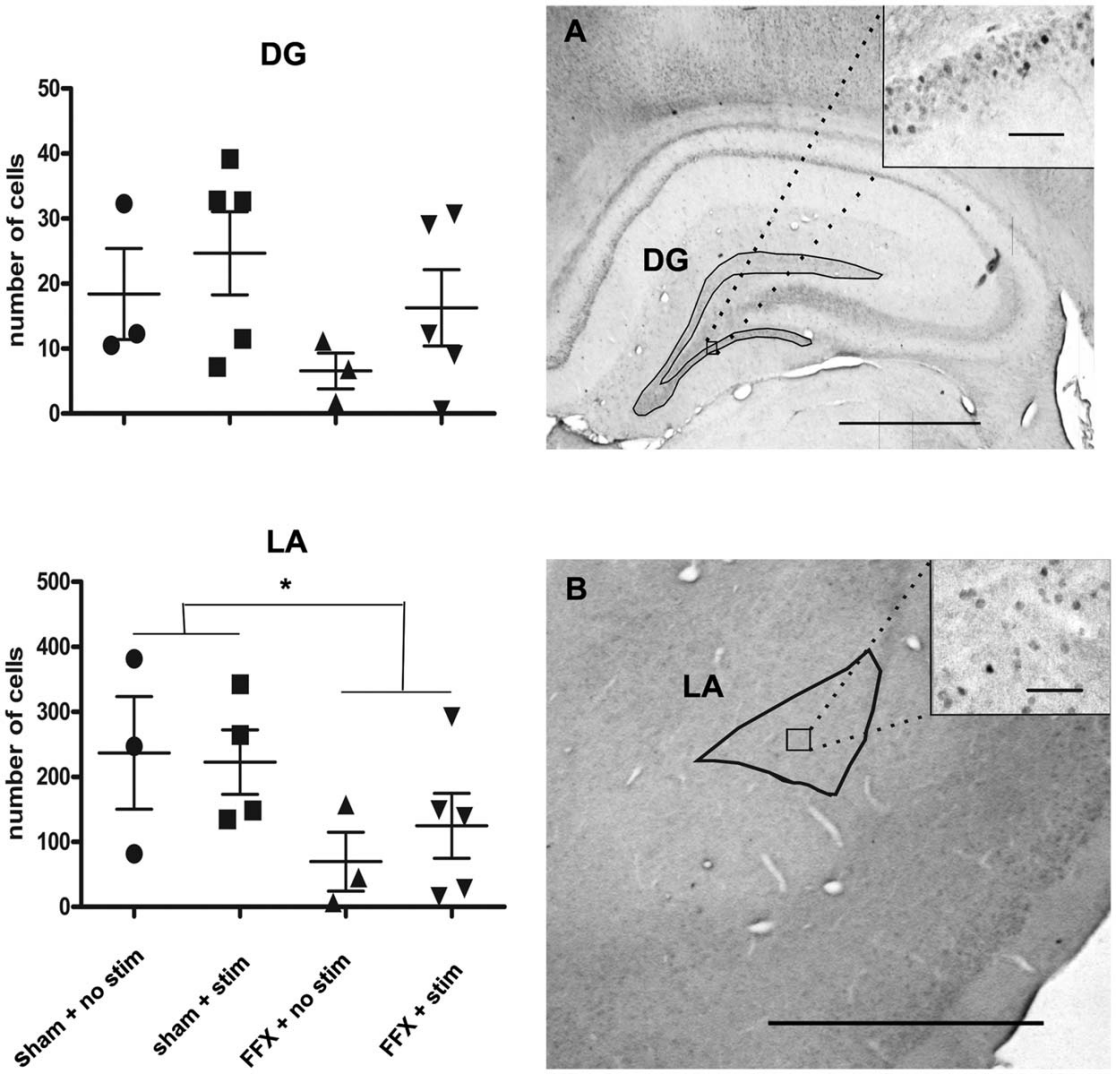
We used the early gene Egr-1 protein product as a molecular marker of the activation of memory consolidation pathways upon re-exposure to the shock context. The number of Egr-1 positive cells was measured in the left dorsal hippocampal dentate gyrus area (DG), in the lateral amygdala (LA) a. The scale bar in the figures A and B represents 1000 µm and in the inserts 200 µm, * *P*<0.05.

To assess induction of molecular pathways involved in memory consolidation, we measured the protein product of the immediate early gene Egr-1 in brain regions most intimately involved in context fear conditioning, the amygdala and hippocampus. In the hippocampus we focused on the dentate gyrus because of the very low constitutive Egr-1 activity in this subregion. We analyzed the effect of lesion and stimulation on the number of Egr-1 positive neurons with a two-way ANOVA. FFX reduced Egr1-positive cells significantly in the amygdala (F_1,11_ = 5.1, p = 0.046; Fig. 6), while the main effect of stimulation or lesion by stimulation interaction were nonsignificant. A similar trend was seen in the dentate gyrus, but that was nonsignificant (F_1,12_ = 2.5, p = 0.14; Fig. 6).

## Discussion

The present experiment set out to directly test the idea that hippocampal theta activity *per se* is necessary for successful memory encoding by assessing whether artificial theta stimulation could restore or at least alleviate impaired contextual fear memory in fimbria-fornix transected rats. We indeed could demonstrate significant memory impairment in the contextual fear conditioning task due to FFX, which was also reflected in a reduced number of Egr-1 positive neurons in the lateral amygdala upon re-exposure to the conditioned context. However, contrary to our predictions, the ATS did not restore the contextual fear memory in FFX rats and in sham-operated rats caused memory impairment that compares with FFX.

As expected, based on the voluminous literature on the critical involvement of the hippocampus in contextual fear conditioning [39]–[41], FFX rats froze less than sham rats in all three test sessions, although the difference became clearly significant only during the second exposure to the familiar environment due to high variability in the groups. The relatively modest effect of FFX cannot be ascribed to incomplete lesion, since judged from the loss of cholinergic terminals all rats included in this study had a complete loss of medial septal inputs to the hippocampus. Furthermore, rats that showed the least amount of conditioning to the context were not the same with the smallest loss of cholinergic input or theta power (data not shown). In addition, FFX resulted in a dramatic loss of hippocampal theta in all FFX rats as described earlier [10], [63], [65], [66]. In fact, whereas retrograde amnesia caused by hippocampal lesion is severe and well established [36], anterograde amnesia as a results of pre-training lesions, as was the case in this study, appear to be mild or even non-existing [39], [67]. In addition, the mild anterograde amnesia related to pre-training lesions seems to be the same in fimbria-fornix, septal hippocampus or entorhinal cortex lesioned animals [39]. Thus, the modest impairment during the first 24-h test session is in keeping with the available literature. The freezing time in a totally novel Context B for both the sham and the lesioned animals was significantly shorter than in Context A1. This suggests that the FFX did not impair the rats’ ability to discriminate between the conditioned Context A and the novel Context B as previously reported [68]. On the other hand, re-exposure to Context A (A2) reduced freezing time of the sham animals as compared to initial testing in Context A (A1), but much more so in FFX rats, resulting in a significant lesion effect. One explanation for this trend is that the initial conditioning induced a weaker memory trace in FFX rats than in sham rats, resulting in accentuated group difference with increasing time and repeated exposures to the shock context. The attenuated freezing response of FFX rats was also associated with blunted amygdalar activation of the early gene Egr 1 compared to sham animals. Since Egr 1 (zif268) has been shown to represent a molecular marker of reconsolidation [47], [48], a careful interpretation of the data is that FFX rats indeed had difficulties in recognizing Context A as the dangerous environment upon second testing.

The artificial theta stimulation (ATS) applied here was successful in restoring the frequency and amplitude of theta to the level of intact rats during movement. Nevertheless, it had no positive effect in the encoding phase of contextual fear. Quite the opposite, the effect of ATS alone in non-lesioned animals resulted in dramatic impairment of contextual fear conditioning that was at least as severe as the effect of FFX. A small further impairment by ATS was also observed in FFX animals. These findings appear at odds with two previous reports on modest improvement by ATS of rats sustaining FFX or medial septal activation in Morris water maze [18], [26]. However, in a more recent study [19] single stimulation pulses delivered at 7.7 Hz did not affect the performance of rats with medial septum inactivation in a radial water maze, while theta-burst stimulation (four-pulse burst 500 Hz-burst at 7.7 Hz) did improve the performance. One obvious reason for the discrepant findings is that our experiment was designed to tap a different memory process than the previous studies. Common to all three earlier ATS studies is the use of some version of water maze and assessment of ATS effect within a session, i.e. affecting a memory process lasting less than an hour. Septohippocampal theta has been involved mainly in visuospatial learning and memory processes. It is clear that water maze is heavily dependent on these processes, whereas fear conditioning in general is not. However, contextual conditioning has been argued to have a hippocampal dependent component, which is thought to represent visuospatial learning of the specific environment before the first foot-shock takes place. Anyway, contextual condition is likely less dependent on visuospatial learning than water maze, which may explain why theta restoration did not have any positive effect in our experiment while some positive results were observed in Morris water maze. In addition, the water maze paradigms did not make a distinction between encoding and retrieval. In contrast, we used a memory task where learning takes place during a single session, limited ATS to the encoding phase of the task, and assessed the effect 24–28 h after ATS delivery. A careful conclusion from these still scattered observations is that hippocampus theta may be more strongly involved in working memory and memory retrieval than encoding into long-term memory. Consistent with this suggestion are several findings that emphasize the involvement of hippocampal theta and its interactions with the frontal cortex in working memory, both in rodents [69] and in humans [70]. In contrast, the data linking hippocampal theta to long-term memory encoding are sparse. The main support to this idea comes from the observation that patterned stimulation of hippocampal inputs results in LTP both *in vitro* [71] and *in vivo* [72] when applied during the positive phase of theta, while the same stimulation during the negative theta phase leads to LTD. Whether the same applies for natural stimulation is largely unknown. In support of this idea, synchronized activity at theta frequencies increased between the lateral nucleus of the amygdala and the CA1 region of the hippocampus after fear conditioning in the rat [43]. However, a later study from the same laboratory specified the occurrence of synchronized hippocampal – amygdalar theta during the retrieval phase 24 h after the fear conditioning [44]. Thus it remains a testable hypothesis that ATS, when specifically delivered during or just before testing of retrieval of fear memory, would restore freezing in FFX animals [73]. This experiment will be challenging to conduct, however, because memory retrieval has to be separated in time from possible direct effects of the theta stimulation.

Several additional factors may also account for the lack of beneficial effect of ATS in the present experiment. First, it should be noted that the FFX not only depleted the hippocampus from the septal pacemakers but also destroyed the main descending output from the hippocampus to the mamillary nuclei. Therefore, the artificial theta induced in the hippocampus itself did not necessarily spread further to the mamillo-thalamic tract. However, one earlier study reported beneficial effect of ATS in the water maze in rats sustaining FFX, which argues against a crucial role of theta restoration in the fornical efferents from the hippocampus [26]. Second, unlike the water maze paradigms employed in the earlier studies, fear conditioning in separate sessions may be susceptible for state dependency. Whereas initial exploration in the stimulated groups took place during ATS, there was no ATS during retrieval.

It still remains enigmatic why ATS during the encoding phase of contextual fear conditioning resulted in significant impairment of memory more than 24 hours later. One conventional explanation for the ATS induced impairment, especially in the sham animals, is the above mentioned state dependency. Maybe more important, however, is the fact that electrical stimulation of ventral hippocampal commisure forces all hippocampal neurons to follow a single theta oscillator. It is well established that hippocampal theta is driven by rhythm generators in the medial septum and entorhinal cortex [5]. In addition, recent studies have revealed also hippocampal intrinsic circuitry or multiple cell assemblies which give rise to a self-organized theta rhythm [17], [74], [75]. Perhaps ATS is not able to substitute the abolished natural complex electrophysiological activity, and therefore, forcing all hippocampal sub-regions to follow the same external theta prevents such formation of task-specific cell assemblies.

Another, and maybe an even more important aspect, is that exogenous theta stimulation we used was fixed in its frequency and totally uncoupled to the rat’s own behavior. Usually walking [76] and sniffing [77] in a new environment get entrained with hippocampal theta during active exploration in a rat, which may set favorable conditions for weak environmental stimuli to elicit synaptic changes as happens during artificial theta-patterned LTP [71], [72]. In contrast, if the external theta stimulation is delivered out of synchrony to these rat-initiated activities, the result can be a weakening of the encoded memory trace. In support to this notion, the only study so far applying yoked (‘by-pass’) theta after septal inactivation demonstrated better enhancement of spatial learning with this ATS than with stimulation using a fixed 7.7 Hz frequency [18]. It would be interesting to see in future experiments, whether ATS when synchronized with an external cue (for instance a tone in a trace conditioning paradigm) would actually enhance the formation of long-term memory trace.

The positive outcome of the present study is the demonstration that ATS indeed can affect not only short-term working memory but also long-term memory. Surprisingly,this time memory was impaired. There are some encouraging clinical reports that stimulation of fimbria-fornix [21], [23] or the nucleus basalis of Meynert [22] activated the hippocampus and enhanced cognitive functions in human patients with memory impairment. However, it is at the same time clear that more research is needed to find the optimal stimulation parameters in terms of the pathway to stimulate and the stimulation pattern to use. In addition, a relevant question is which memory phase is the best one to apply such stimulation (encoding, early consolidation just after the learning episode, or retrieval). As demonstrated in the present study, fear conditioning, by providing a clear separation between various stages of memory formation, would offer an excellent model to test stimulation effect in these different stages. A severe disadvantage of electrical stimulation of the brain tissue is the nonspecific activation of the neuronal cell groups in the target area [78]. In this regard, the rapid recent advancement in optogenetic techniques offering more precise timing and more cell type-specific actions by external stimulation, could improve the outcome of the stimulation treatment in future experiments [79].
